# Supplementary material for: 131 genetic loci highlight immunological pathways and tissues in nasal polyposis and asthma
Source: Nat Commun. 2025 Nov 10;16:9879. doi: 10.1038/s41467-025-64847-4 (PMC12603121; doi:10.1038/s41467-025-64847-4)
Supplement: Supplementary file 1 — Supplementary Information [file 41467_2025_64847_MOESM1_ESM.pdf]

131 genetic loci highlight immunological pathways and tissues in nasal polyposis and asthma

Supplementary Information

Contents

Supplementary Note 1 2

Supplementary Tables 3

Supplementary Figures 10

Supplementary information

Multi-morbidity is a traditional problem in statistics, increasingly a problem of epidemiological studies in aging populations. Analysing diseases independently implies that the diseases can be considered. Common alternatives are multivariate modelling, or Bayesian hierarchies as used here.

To investigate the challenge of co-morbidity, we also analysed the impact of selecting cases exclusively with one disorder in FinnGen R9, and ran their genome-wide association. We then compared these to our non-exclusive, "original" analyses.

Asthma without co-morbid CRS was observed for 36,116 of 42,163 participants (85.7 %), and a GWAS of these observed 30 loci, vs 33 in original FinnGen analysis. One of the 30 loci, at 3p24.1, is not observed in original FinnGen analysis. Variants at this locus were mostly absent in UKB, or showed no association ($p > 0.05$).

CRSwNP cases without comorbid asthma was observed for 3919 of 6255 cases (62.6 %), and a GWAS of these detected 12 genomic loci, vs 30 in original FinnGen analysis. 11 of the 12 loci is observed in the original FinnGen analysis, and one (6p25.3b) is not GWS in original analysis, but is observed in meta-analysis.

CRSsNP cases without asthma was observed for 9823 of 13,534 participants (72.6 %) and highlighting 4 loci vs 8 in the original FinnGen analysis. One of the 4 loci, at cytoband 2q14.1, is not observed in the original analysis, and contains a single rare variant ($AF < 0.1$ %) that is not tested for in the UKB.

Using a cross-trait analysis that only considers participants with all three diagnoses (an intersection of all three) highlights 13 genomic loci, compared with 49 genomic loci when a union of all three is considered.

Notably, despite analyzing with an exclusive approach, we detect loci 5q22.1b (near *TSLP*) and 9p24.1 (near *IL33*) in all four analyses, and 10p14b (near *GATA3*) in both asthma and CRSwNP, albeit with non-comparable effect estimates. These results demonstrate a loss of power in the categorical approach.

Supplementary Tables

Supplementary Table 1:Genome-wide association study metrics and summary statistics. λ_{GC} is the genomic inflation factor; "Intercept" denotes the LD Score regression intercept; and "Intercept SE" denotes its standard error. $\hat{h}^2_{SNP}$ denotes the SNP-based heritability estimate derived from LD Score regression, and converted to liability scale. Population frequency estimates for observed-to-liability transformation were set at K=0.15 for asthma, K=0.04 for CRSwNP, and K=0.035 for CRSsNP. "$\hat{h}^2_{SNP}$ 95 % CI" denotes a 95 % confidence interval, first calculated at the observed scale and converted to the liability scale.

Phenotype	Cohort	λ_{GC}	Intercept	Intercept SE	$\hat{h}^2_{SNP}$	$\hat{h}^2_{SNP}$ 95 % CI
Asthma	UKB	1.23	1.04	0.0093	17.1 %	14.6 %–19.6 %
Asthma	FinnGen	1.38	1.14	0.0124	13.7 %	11.5 %–16.0 %
Asthma	Meta-analysis	1.52	1.12	0.013	N/A	N/A
CRSwNP	UKB	1.06	1.01	0.0076	24.1 %	14.8 %–33.4 %
CRSwNP	FinnGen	1.16	1.06	0.0109	33.2 %	25.3 %–41.1 %
CRSwNP	Meta-analysis	1.17	1.05	0.0106	N/A	N/A
CRSsNP	UKB	1.01	1.00	0.0067	7.2 %	-2.9 %–17.3 %
CRSsNP	FinnGen	1.13	1.04	0.0075	6.6 %	5.0 %–8.1 %
CRSsNP	Meta-analysis	1.13	1.04	0.0071	N/A	N/A
Asthma and/or CRS	UKB	1.22	1.04	0.0094	N/A	N/A
Asthma and/or CRS	FinnGen	1.38	1.13	0.0124	N/A	N/A
Asthma and/or CRS	Meta-analysis	1.49	1.12	0.0135	N/A	N/A

Supplementary Table 2: Tissues significantly enriched in MAGMA tissue enrichment analysis. Phenotype: phenotype of GWAS used in analysis. Tissue: GTEx v8 tissue from which expression data is derived (54 tissues). β_{STD}*: standardized beta from analysis. SE: standard error. p: p-value from* χ^2 *distribution with one degree of freedom. Only tissues with significant enrichment after multiple testing correction are included (p < 3.09E-04); no significant enrichment was observed for CRSwNP.*

Phenotype	Tissue	β_{STD}	SE	p
Asthma	EBV-transformed lymphocytes	0.0487	0.0055	2.17E-05
Asthma	Spleen	0.0690	0.0082	1.04E-05
Asthma	Whole blood	0.0423	0.0063	8.57E-05
Asthma and/or CRS	Spleen	0.0732	0.0081	2.43E-06
Asthma and/or CRS	Whole blood	0.0433	0.0062	5.26E-05

Supplementary Table 3: Genetic correlation analyses using LD Score Regression. Phenotype #: GWAS phenotype and cohort, such that FG = FinnGen R9, and UK = UK Biobank. r_G = genetic correlation coefficient. r_G 95 % CI = 95 % confidence interval for genetic correlation coefficient (maximum is 100 %). p = p-value of correlation under x2 approximation (one degree of freedom). CRSsNP (UK) had a non-significant heritability under LD Score Regression and was excluded from these analyses.

Phenotype 1	Phenotype 2	r_G	r_G 95 % CI	p
Asthma (FG)	Asthma (UK)	86.5 %	80.6 %–92.3 %	7.04E-185
CRSwNP (FG)	CRSwNP (UK)	91.8 %	75.8 %–100.0 %	1.66E-29
Asthma (FG)	CRSwNP (FG)	52.7 %	42.7 %–62.7 %	4.43E-25
Asthma (UK)	CRSwNP (UK)	61.6 %	48.6 %–74.7 %	2.27E-20
Asthma (FG)	CRSsNP (FG)	63.3 %	52.3 %–74.2 %	1.56E-29
CRSwNP (FG)	CRSsNP (FG)	59.3 %	46.9 %–71.7 %	8.80E-21

Supplementary Table 4: FinnGen study approvals.

Institution	Permit number
Finnish Institute for Health and Welfare (THL)	THL/2031/6.02.00/2017
Finnish Institute for Health and Welfare (THL)	THL/1101/5.05.00/2017
Finnish Institute for Health and Welfare (THL)	THL/341/6.02.00/2018
Finnish Institute for Health and Welfare (THL)	THL/2222/6.02.00/2018
Finnish Institute for Health and Welfare (THL)	THL/283/6.02.00/2019
Finnish Institute for Health and Welfare (THL)	THL/1721/5.05.00/2019
Finnish Institute for Health and Welfare (THL)	THL/1524/5.05.00/2020
Finnish Institute for Health and Welfare (THL)	THL/2364/14.02/2020
Finnish Institute for Health and Welfare (THL)	THL/4055/14.06.00/2020
Finnish Institute for Health and Welfare (THL)	THL/3433/14.06.00/2020
Finnish Institute for Health and Welfare (THL)	THL/4432/14.06/2020
Finnish Institute for Health and Welfare (THL)	THL/5189/14.06/2020
Finnish Institute for Health and Welfare (THL)	THL/5894/14.06.00/2020
Finnish Institute for Health and Welfare (THL)	THL/6619/14.06.00/2020
Finnish Institute for Health and Welfare (THL)	THL/209/14.06.00/2021
Finnish Institute for Health and Welfare (THL)	THL/688/14.06.00/2021
Finnish Institute for Health and Welfare (THL)	THL/1284/14.06.00/2021
Finnish Institute for Health and Welfare (THL)	THL/1965/14.06.00/2021
Finnish Institute for Health and Welfare (THL)	THL/5546/14.02.00/2020
Finnish Institute for Health and Welfare (THL)	THL/2658/14.06.00/2021
Finnish Institute for Health and Welfare (THL)	THL/4235/14.06.00/202
Digital and population data service agency (VRK)	VRK43431/2017-3
Digital and population data service agency (VRK)	VRK/6909/2018-3
Digital and population data service agency (VRK)	VRK/4415/2019-3
the Social Insurance Institution (KELA)	KELA 58/522/2017
the Social Insurance Institution (KELA)	KELA 131/522/2018
the Social Insurance Institution (KELA)	KELA 70/522/2019
the Social Insurance Institution (KELA)	KELA 98/522/2019
the Social Insurance Institution (KELA)	KELA 138/522/2019
the Social Insurance Institution (KELA)	KELA 2/522/2020
the Social Insurance Institution (KELA)	KELA 16/522/2020
Statistics Finland	TK-53-1041-17
Statistics Finland	TK/143/07.03.00/2020
Statistics Finland	TK/1735/07.03.00/2021
Statistics Finland	TK/3112/07.03.00/2021

Supplementary Table 5: FinnGen study Biobank access decisions

**Biobank**	**Accession number**
THL Biobank	BB2017_55
THL Biobank	BB2017_111
THL Biobank	BB2018_19
THL Biobank	BB_2018_34
THL Biobank	BB_2018_67
THL Biobank	BB2018_71
THL Biobank	BB2019_7
THL Biobank	BB2019_8
THL Biobank	BB2019_26
THL Biobank	BB2020_1
Finnish Red Cross Blood Service Biobank	7.12.2017
Helsinki Biobank	HUS/359/2017
Helsinki Biobank	HUS/248/2020
Auria Biobank	AB17-5154 and amendment #1 (Aug 17, 2020)
Auria Biobank	AB20-5926 and amendment #1 (Apr 23, 2020) and modification (Sep 22, 2021)
Biobank Borealis of Northern Finland	Biobank Borealis of Northern Finland_2017_1013
Biobank of Eastern Finland	1186/2018 and amendment 22 § /2020
Finnish Clinical Biobank Tampere	MH0004 and amendments (Feb 21, 2020 and Oct 6, 2020)
Central Finland Biobank	1-2017
Terveystalo Biobank	STB 2018001 and amendment (Aug 25, 2020)

Supplementary Table 6: Epidemiological overlaps of cases in FinnGen R9. Diagonal corresponds to full case count.

FinnGen	Asthma cases	CRSwNP cases	CRSsNP cases
…with asthma	42163	2336	3711
…with CRSwNP	2336	6255	0
…with CRSsNP	3711	0	13534

Supplementary Table 7: Epidemiological overlaps of cases in the UK Biobank. Diagonal corresponds to full case count.

UKB	Asthma cases	CRSwNP cases	CRSsNP cases
…with asthma	29318	1185	358
…with CRSwNP	1185	3371	0
…with CRSsNP	358	0	1914

Supplementary Figures

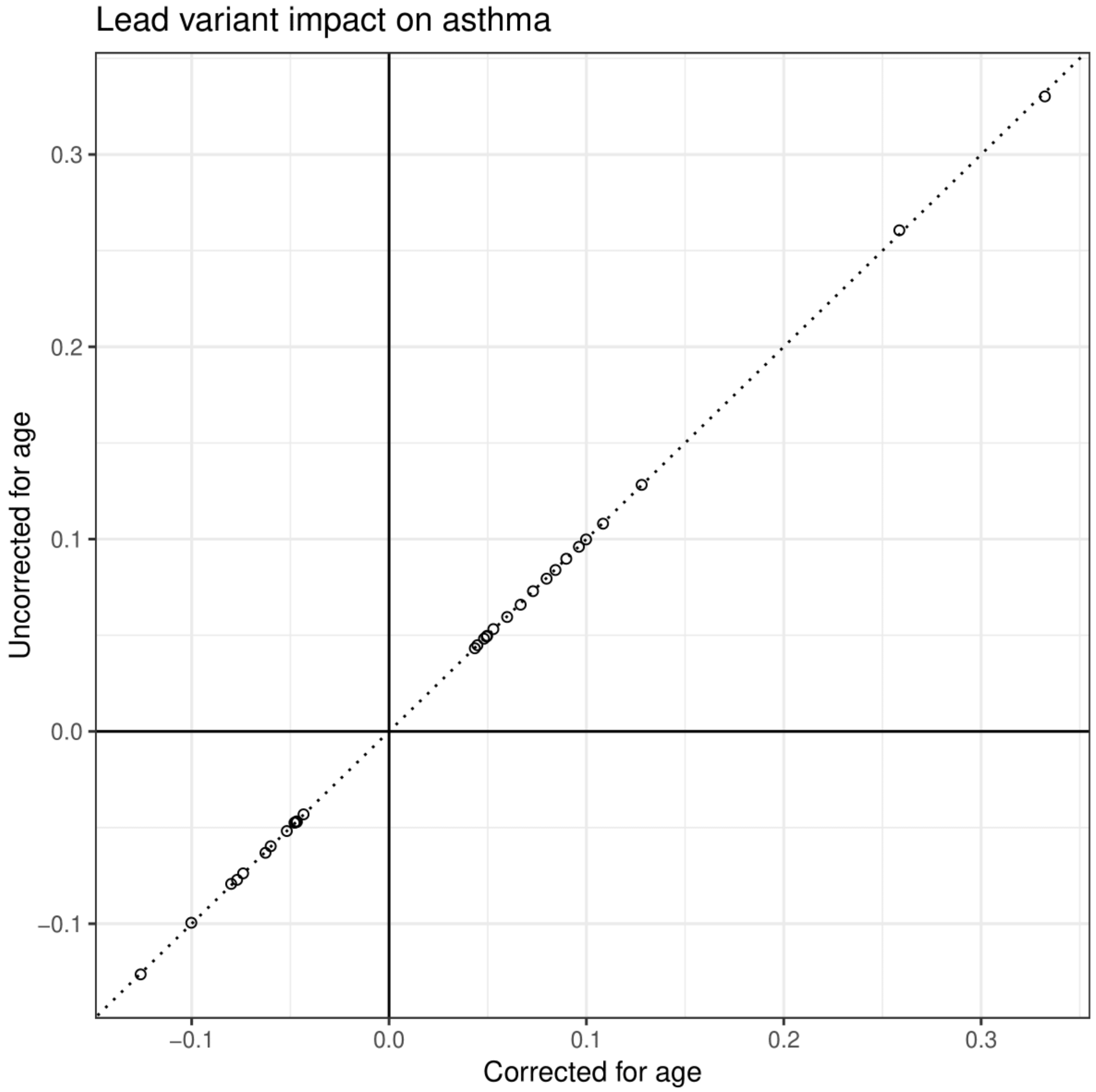

Supplementary Figure 1: Lead variants associated with asthma in FinnGen R9 (42,163 cases vs 321,372 controls) with and without age correction. Genome-wide analysis was run using REGENIE and corrected for age at end of follow-up, sex and PCs 1–10 (horizontal), or corrected only for sex and PCs 1–10 (vertical. Variant association (log OR) remains near-exactly on a y=x trendline (dotted) despite removal of age as covariate. Log OR is derived from a right-tailed χ^2-distribution with one degree of freedom in both cases.

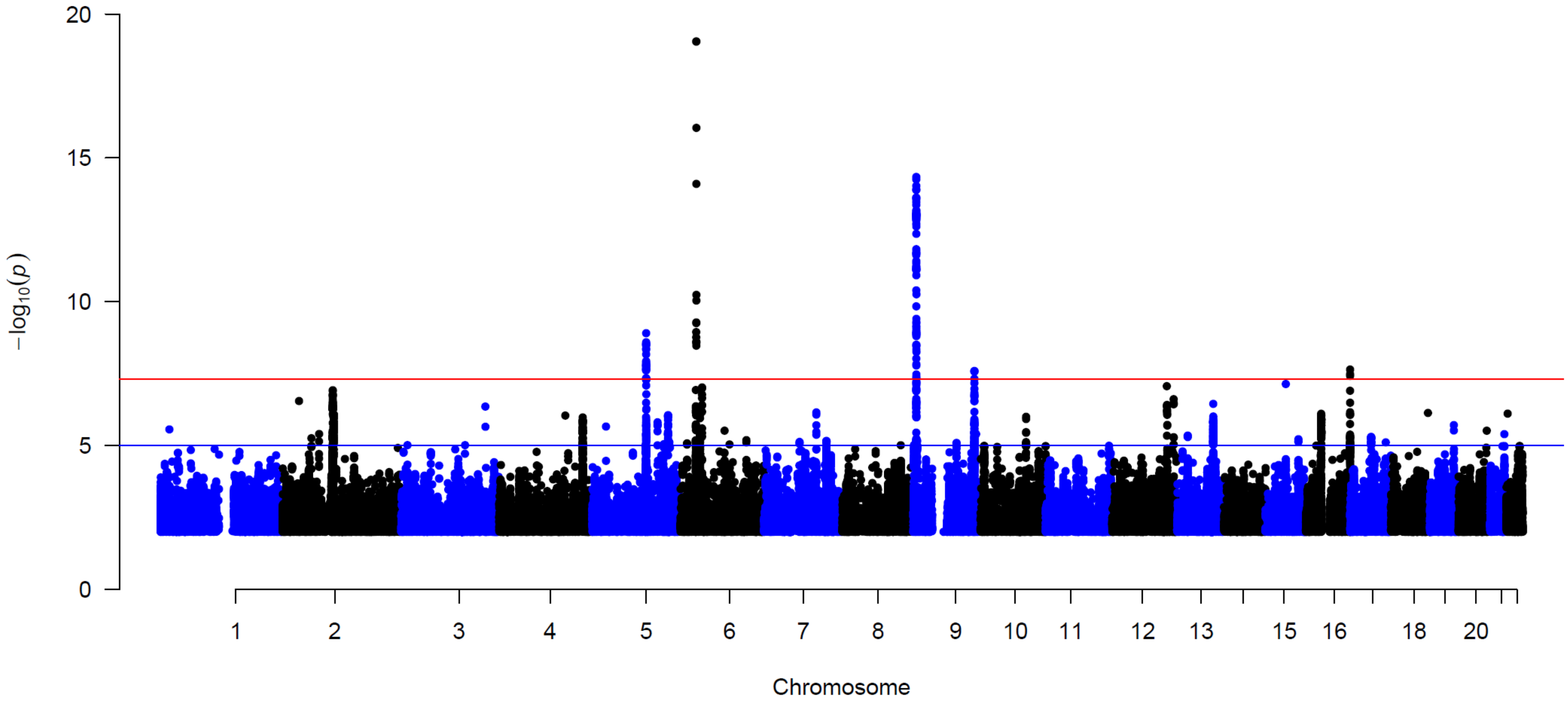

Supplementary Figure 2: Manhattan plot of genome-wide meta-analysis results for association of 7,268,979 variants with CRSsNP in FinnGen and UKB (13,534 cases vs 685,602 controls). Vertical axis denotes negative log10 p-values of variants, and horizontal axis represents the genomic position by chromosome (hg38 coordinates). The red dashed line denotes genome-wide significance (p<5e-8). P-values are derived from one-sided χ^2-distribution with one degree of freedom. Lead variants of the five genome-wide significant loci are described in Supplementary Data 3.

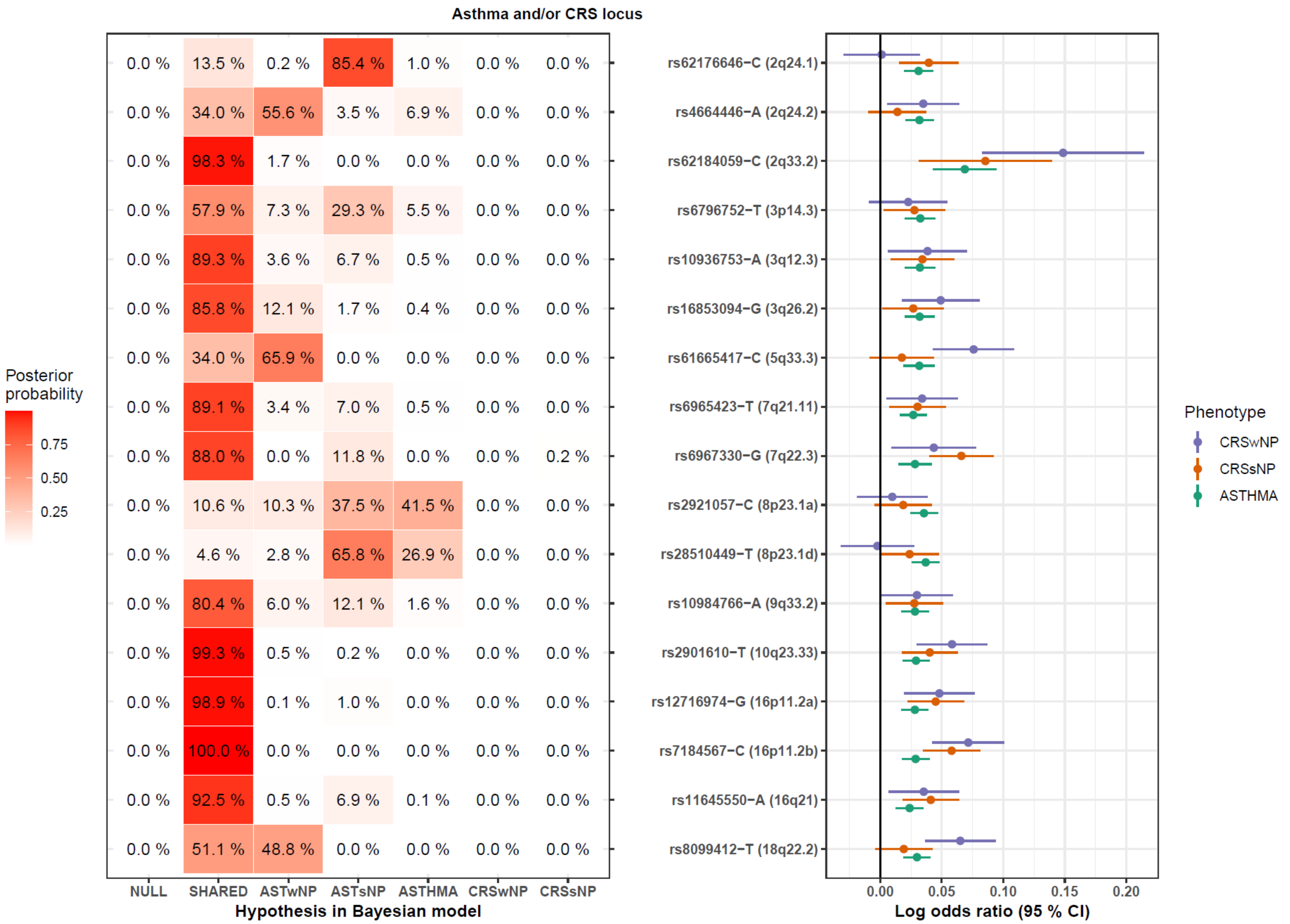

NULL	SHARED	ASTwNP	ASTsNP	ASTHMA	CRSwNP	CRSsNP	Locus
0.0 %	13.5 %	0.2 %	85.4 %	1.0 %	0.0 %	0.0 %	rs62176646−C (2q24.1)
0.0 %	34.0 %	55.6 %	3.5 %	6.9 %	0.0 %	0.0 %	rs4664446−A (2q24.2)
0.0 %	98.3 %	1.7 %	0.0 %	0.0 %	0.0 %	0.0 %	rs62184059−C (2q33.2)
0.0 %	57.9 %	7.3 %	29.3 %	5.5 %	0.0 %	0.0 %	rs6796752−T (3p14.3)
0.0 %	89.3 %	3.6 %	6.7 %	0.5 %	0.0 %	0.0 %	rs10936753−A (3q12.3)
0.0 %	85.8 %	12.1 %	1.7 %	0.4 %	0.0 %	0.0 %	rs16853094−G (3q26.2)
0.0 %	34.0 %	65.9 %	0.0 %	0.0 %	0.0 %	0.0 %	rs61665417−C (5q33.3)
0.0 %	89.1 %	3.4 %	7.0 %	0.5 %	0.0 %	0.0 %	rs6965423−T (7q21.11)
0.0 %	88.0 %	0.0 %	11.8 %	0.0 %	0.0 %	0.2 %	rs6967330−G (7q22.3)
0.0 %	10.6 %	10.3 %	37.5 %	41.5 %	0.0 %	0.0 %	rs2921057−C (8p23.1a)
0.0 %	4.6 %	2.8 %	65.8 %	26.9 %	0.0 %	0.0 %	rs28510449−T (8p23.1d)
0.0 %	80.4 %	6.0 %	12.1 %	1.6 %	0.0 %	0.0 %	rs10984766−A (9q33.2)
0.0 %	99.3 %	0.5 %	0.2 %	0.0 %	0.0 %	0.0 %	rs2901610−T (10q23.33)
0.0 %	98.9 %	0.1 %	1.0 %	0.0 %	0.0 %	0.0 %	rs12716974−G (16p11.2a)
0.0 %	100.0 %	0.0 %	0.0 %	0.0 %	0.0 %	0.0 %	rs7184567−C (16p11.2b)
0.0 %	92.5 %	0.5 %	6.9 %	0.1 %	0.0 %	0.0 %	rs11645550−A (16q21)
0.0 %	51.1 %	48.8 %	0.0 %	0.0 %	0.0 %	0.0 %	rs8099412−T (18q22.2)

Supplementary Figure 3: Bayesian cross-trait analysis of lead variants of loci not detected in asthma or CRS analyses. A (left): Bayesian analysis indicating the most probable model. NULL: Null model. SHARED: the effect is identical or very similar for both asthma and CRS. ASTwNP: variant has similar effect on ASTHMA and CRSwNP, none for CRSsNP. ASTsNP: variant has similar effect on ASTHMA and CRSsNP, no effect on CRSwNP. ASTHMA: variant impacts asthma only. CRSwNP: variant impacts CRSwNP only. CRSsNP: variant impacts CRSsNP only. B (right): Forest plot of odds ratio with 95 % confidence intervals (+/- 1.96 SE) of lead variants in phenotype-specific analyses.

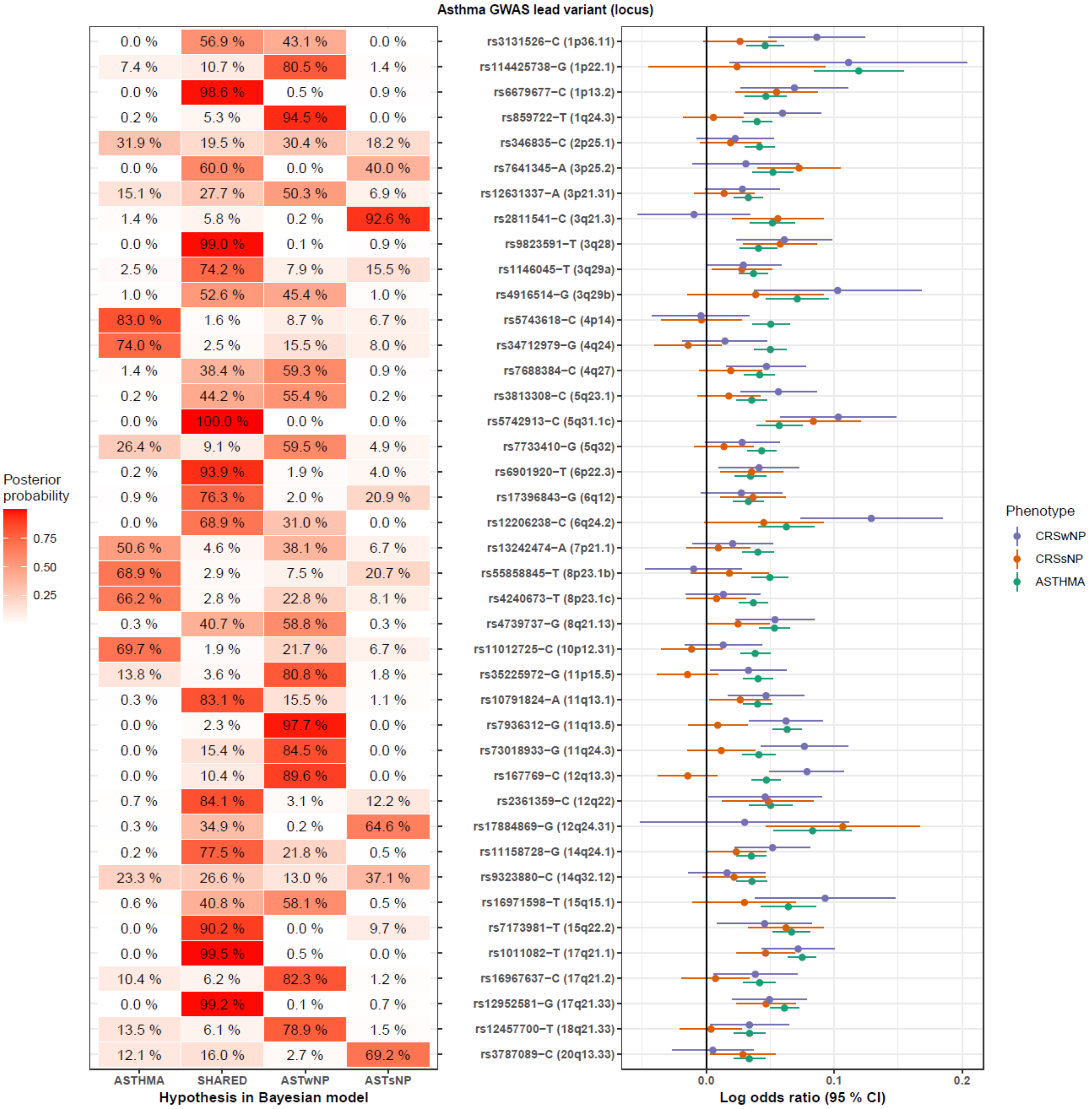

Supplementary Figure 4: Shared effects in Bayesian cross-trait analysis of lead variants seen in the FinnGen-UKB asthma GWAS but not CRSwNP GWAS. A (left): Bayesian analysis indicating the most probable model. ASTHMA: there is an effect on asthma but no effect on CRSwNP; SHARED: the effect is identical or very similar in both asthma and CRS; ASTwNP: variant has similar effect on ASTHMA and CRSwNP, none for CRSsNP; ASTsNP: variant has similar effect on ASTHMA and CRSsNP, no effect on CRSwNP.
B (right): Forest plot of log odds ratio with 95 % confidence intervals (+/- 1.96 SE) of lead variants in phenotype-specific analyses.

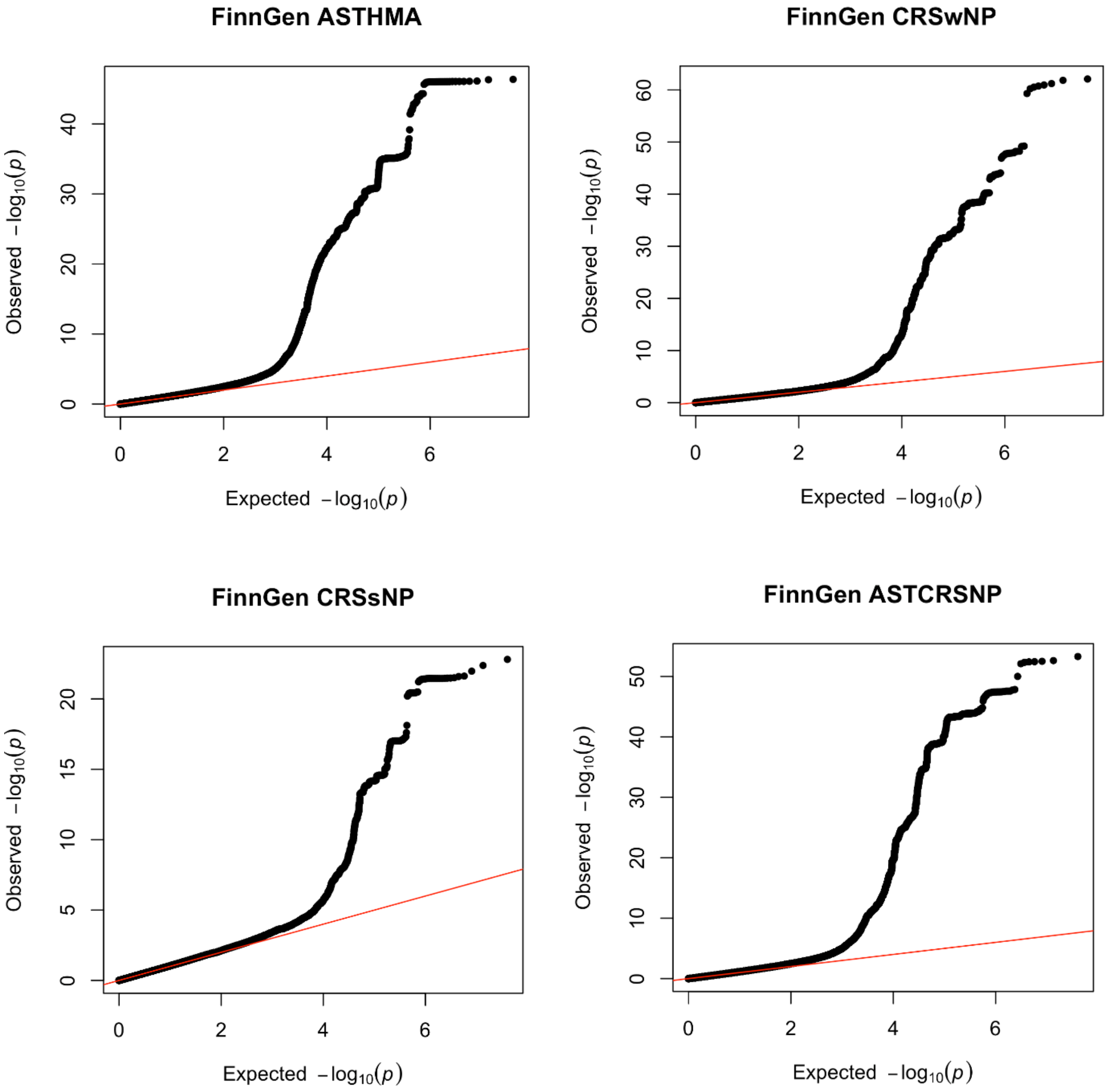

Supplementary Figure 5: Quantile-quantile plots for genome-wide association studies performed in FinnGen R9. Vertical axis is observed p-value frequency, and horizontal axis is expected p-value frequency (χ^2-distribution with one degree of freedom). Red is equal frequency trendline. Top left: Asthma GWAS (42,163 cases vs 321,372 controls). Top right: CRSwNP GWAS (6255 cases vs 321,372 controls). Bottom left: CRSsNP GWAS (13,534 cases vs 321,372 controls). Bottom right: Asthma and/or CRS GWAS (55,905 cases vs 321,372 controls). Early adherence to trendline in all plots suggests true polygenicity for all GWAS.

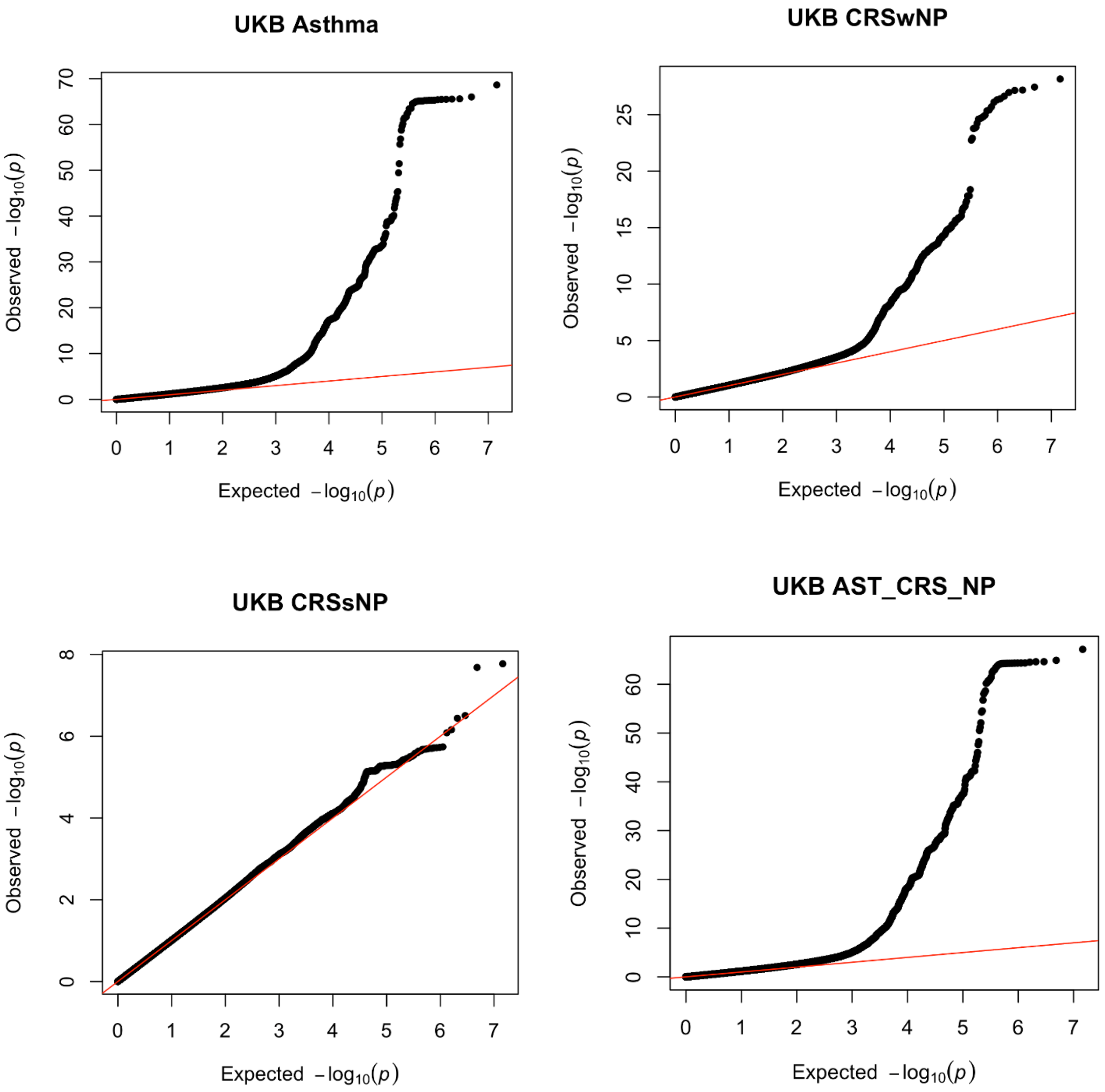

Supplementary Figure 6: Quantile-quantile plots for genome-wide association studies performed in the UK Biobank. Vertical axis is observed p-value frequency, and horizontal axis is expected p-value frequency. Red is equal frequency trendline. Top left: Asthma GWAS (29,318 cases vs 364,230 controls). Top right: CRSwNP GWAS (3371 cases vs 364,230 controls). Bottom left: CRSsNP GWAS (1914 cases vs 364,230 controls). Bottom right: Asthma and/or CRS GWAS (33,060 cases vs 364,230 controls). Early adherence to trendline suggests true polygenicity for all GWAS except CRSsNP, where little deviation from expected values suggests no significant signal.

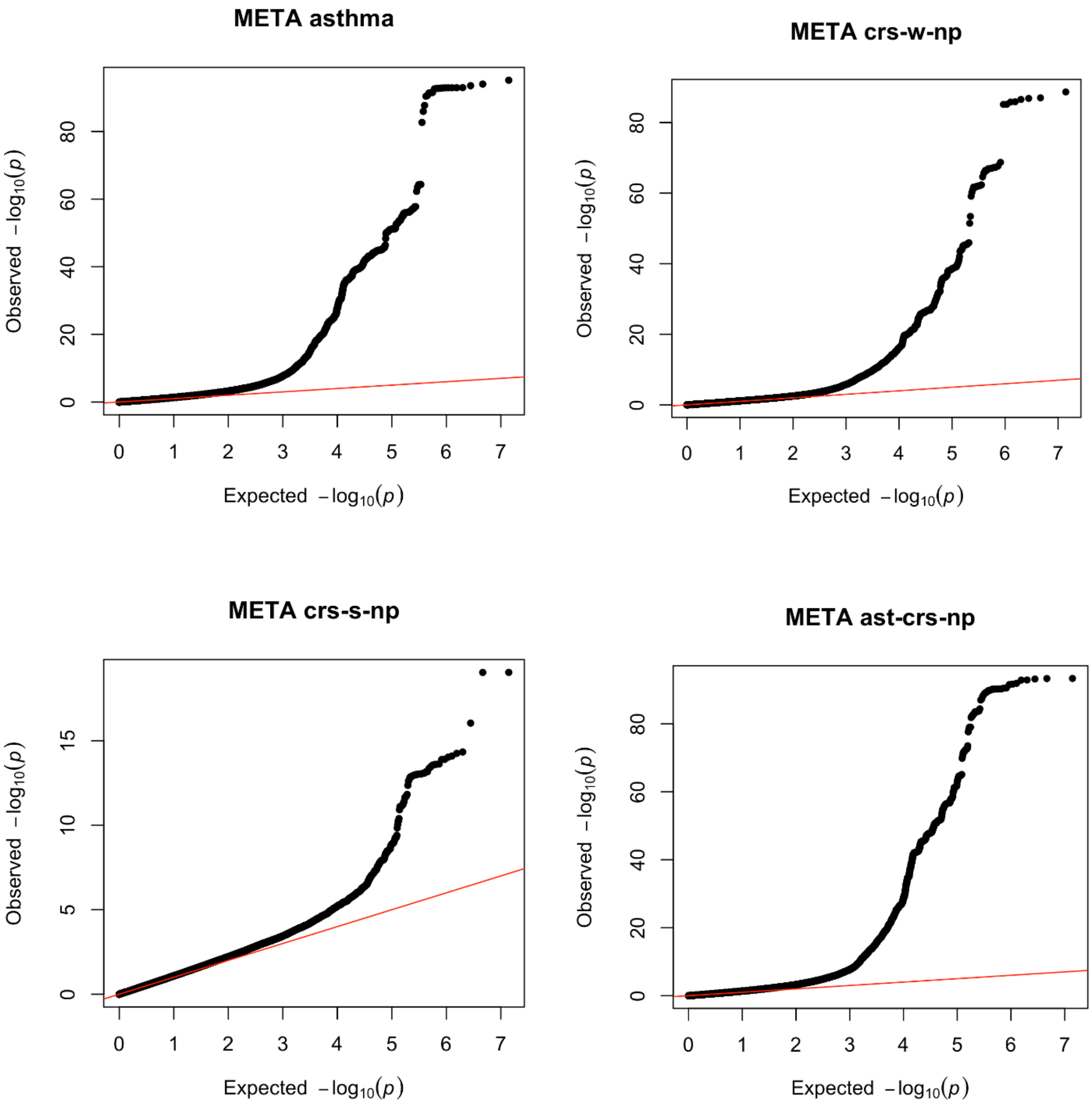

Supplementary Figure 7: Quantile-quantile plots for genome-wide association study meta-analyses. Vertical axis is observed p-value frequency, and horizontal axis is expected p-value frequency. Red is equal frequency trendline. Top left: Asthma GWAS (71,481 cases vs 685,602 controls). Top right: CRSwNP GWAS (9626 cases vs 685,602 controls). Bottom left: CRSsNP GWAS (15,448 cases vs 685,602 controls). Bottom right: Asthma and/or CRS GWAS (88,965 cases vs 685,602 controls). Early adherence to trendline with deviation in all plots suggests true polygenicity and mild degree of inflation.
