# Supplementary material for: 131 genetic loci highlight immunological pathways and tissues in nasal polyposis and asthma
Source: Nat Commun. 2025 Nov 10;16:9879. doi: 10.1038/s41467-025-64847-4 (PMC12603121; doi:10.1038/s41467-025-64847-4)
Supplement: Supplementary file 2 — Description of Additional Supplementary Files [file 41467_2025_64847_MOESM2_ESM.pdf]

# Description of Additional Supplementary Files

Supplementary Data 1: Asthma GWAS loci lead variants. Lead variants of independent genomic loci associated with asthma in meta-analysis of FinnGen and UKB analyses.

Supplementary Data 2: CRSwNP GWAS loci lead variants. Lead variants of independent genomic loci associated with CRSwNP in meta-analysis of FinnGen and UKB analyses.

Supplementary Data 3: CRSsNP GWAS loci lead variants. Lead variants of independent genomic loci associated with CRSsNP in meta-analysis of FinnGen and UKB analyses.

Supplementary Data 4: Asthma and/or CRS cross-trait GWAS loci lead variants. Lead variants of independent genomic loci associated with asthma and/or CRS (combined phenotype) in meta-analysis of FinnGen and UKB analyses.

Supplementary Data 5: Loci characterizations and shared phenotype analysis. Lead variants of independent genomic loci associated with any of the tested in phenotypes in meta-analysis of FinnGen and UKB analyses.

Supplementary Data 6: Credible set variants from Asthma GWAS in FinnGen R9. Variants of credible sets finemapped with SuSIE in the FinnGen (Release 9) genome-wide association of asthma.

Supplementary Data 7: Credible set variants from CRSwNP GWAS in FinnGen R9. Variants of credible sets finemapped with SuSIE in the FinnGen (Release 9) genome-wide association of CRSwNP.

Supplementary Data 8: Credible set variants from CRSsNP GWAS in FinnGen R9. Variants of credible sets finemapped with SuSIE in the FinnGen (Release 9) genome-wide association of CRSsNP.

Supplementary Data 9: Credible set variants from Asthma and/or CRS cross-trait GWAS in FinnGen R9. Variants of credible sets finemapped with SuSIE in the FinnGen (Release 9) genome-wide association of asthma and/or CRS (combined phenotype analysis).

Supplementary Data 10: Colocalization results of credible sets with select FinnGen endpoints. Colocalization of identified credible sets of FinnGen loci with select FinnGen endpoints. Colocalization analysis run by in-house pipeline powered by eCAVIAR.

Supplementary Data 11: Colocalization results of credible sets with eQTLs of 44 tissues in GTEx. Colocalization of identified credible sets of FinnGen loci with select tissues as provided by GTEx. Colocalization analysis run by in-house pipeline powered by eCAVIAR.

Supplementary Data 12: MAGMA gene enrichment results. Identified genes with variant enrichment when performing MAGMA analysis.

Supplementary Data 13: MAGMA gene set analysis results. Gene sets with enriched genes identified with MAGMA software.

Supplementary Data 14: Genomic loci eQTL analysis results. Reported eQTL enrichment in GTEx of lead variants of all identified loci.

Supplementary Data 15: Full list of FinnGen authors and their affiliations
